# Pixel-Based Machine Learning in Medical Imaging

**DOI:** 10.1155/2012/792079

**Published:** 2012-02-28

**Authors:** Kenji Suzuki

**Affiliations:** Department of Radiology, The University of Chicago, 5841 South Maryland Avenue, MC 2026, Chicago, IL 60637, USA

## Abstract

Machine learning (ML) plays an important role in the medical imaging field, including medical image analysis and computer-aided diagnosis, because objects such as lesions and organs may not be represented accurately by a simple equation; thus, medical pattern recognition essentially require “learning from examples.” One of the most popular uses of ML is classification of objects such as lesions into certain classes (e.g., abnormal or normal, or lesions or nonlesions) based on input features (e.g., contrast and circularity) obtained from segmented object candidates. Recently, pixel/voxel-based ML (PML) emerged in medical image processing/analysis, which use pixel/voxel values in images directly instead of features calculated from segmented objects as input information; thus, feature calculation or segmentation is not required. Because the PML can avoid errors caused by inaccurate feature calculation and segmentation which often occur for subtle or complex objects, the performance of the PML can potentially be higher for such objects than that of common classifiers (i.e., feature-based MLs). In this paper, PMLs are surveyed to make clear (a) classes of PMLs, (b) similarities and differences within (among) different PMLs and those between PMLs and feature-based MLs, (c) advantages and limitations of PMLs, and (d) their applications in medical imaging.

## 1. Introduction

Machine learning (ML) plays an essential role in the medical imaging field, including medical image analysis and computer-aided diagnosis (CAD) [1, 2], because objects such as lesions and organs in medical images may be too complex to be represented accurately by a simple equation; modeling of such complex objects often requires a number of parameters which have to be determined by data. For example, a lung nodule is generally modeled as a solid sphere, but there are nodules of various shapes and nodules with internal inhomogeneities, such as spiculated nodules and ground-glass nodules [3]. A polyp in the colon is modeled as a bulbous object, but there are also polyps which exhibit a flat shape [4, 5]. Thus, diagnostic tasks in medical images essentially require “learning from examples (or data)” to determine a number of parameters in a complex model.

One of the most popular uses of ML in medical image analysis is the classification of objects such as lesions into certain classes (e.g., abnormal or normal, lesions or non-lesions, and malignant or benign) based on input features (e.g., contrast, area, and circularity) obtained from segmented object candidates (This class of ML is referred to feature-based ML.). The task of ML here is to determine “optimal” boundaries for separating classes in the multidimensional feature space which is formed by the input features [6]. ML algorithms for classification include linear discriminant analysis [7], quadratic discriminant analysis [7], multilayer perceptron [8, 9], and support vector machines [10, 11]. Such ML algorithms were applied to lung nodule detection in chest radiography [12–15] and thoracic CT [16–19], classification of lung nodules into benign or malignant in chest radiography [20] and thoracic CT [21, 22], detection of microcalcifications in mammography [23–26], detection of masses in mammography [27], classification of masses into benign or malignant in mammography [28–30], polyp detection in CT colonography [31–33], determining subjective similarity measure of mammographic images [34–36], and detection of aneurysms in brain MRI [37].

Recently, as available computational power increased dramatically, pixel/voxel-based ML (PML) emerged in medical image processing/analysis which uses pixel/voxel values in images directly instead of features calculated from segmented regions as input information; thus, feature calculation or segmentation is not required. Because the PML can avoid errors caused by inaccurate feature calculation and segmentation which often occur for subtle or complex objects, the performance of the PML can potentially be higher for such objects than that of common classifiers (i.e., feature-based MLs). In this paper, PMLs are surveyed and reviewed to make clear (a) classes of PMLs, (b) the similarities and differences within different PMLs and those between PMLs and feature-based MLs, (c) the advantages and limitations of PMLs, and (d) their applications in medical imaging.

## 2. Pixel/Voxel-Based Machine Learning (PML)

### 2.1. Overview

PMLs have been developed for tasks in medical image processing/analysis and computer vision.  Table 1 summarizes classes of PMLs, their functions, and their applications. There are three classes of PMLs: neural filters [38, 39] (including neural edge enhancers [40, 41]), convolution neural networks (NNs) [42–48] (including shift-invariant NNs [49–51]), and massive-training artificial neural networks (MTANNs) [52–56] (including multiple MTANNs [17, 38, 39, 52, 57, 58], a mixture of expert MTANNs [59, 60], a multiresolution MTANN [54], a Laplacian eigenfunction MTANN (LAP-MTANN) [61], and a massive-training support vector regression (MTSVR) [62]). The class of neural filters has been used for image-processing tasks such as edge-preserving noise reduction in radiographs and other digital pictures [38, 39], edge enhancement from noisy images [40], and enhancement of subjective edges traced by a physician in left ventriculograms [41]. The class of convolution NNs has been applied to classification tasks such as false-positive (FP) reduction in CAD schemes for detection of lung nodules in chest radiographs (also known as chest X-rays; CXRs) [42–44], FP reduction in CAD schemes for detection of microcalcifications [45] and masses [46] in mammography, face recognition [47], and character recognition [48]. The class of MTANNs has been used for classification, such as FP reduction in CAD schemes for detection of lung nodules in CXR [57] and CT [17, 52, 63], distinction between benign and malignant lung nodules in CT [58], and FP reduction in a CAD scheme for polyp detection in CT colonography [53, 59–62]. The MTANNs have also been applied to pattern enhancement and suppression such as separation of bone from soft tissue in CXR [54, 55] and enhancement of lung nodules in CT [56]. There are other PML approaches in the literature. An iterative, pixel-based, supervised, statistical classification method called iterated contextual pixel classification has been proposed for segmenting posterior ribs in CXR [64]. A pixel-based, supervised regression filtering technique called filter learning has been proposed for separation of ribs from soft tissue in CXR [65].

### 2.2. Neural Filters

In the field of signal/image processing, supervised nonlinear filters based on a multilayer ANN, called neural filters, have been studied [38, 39]. The neural filter employs a linear-output ANN model as a convolution kernel of a filter. The inputs to the neural filter are an object pixel value and spatially/spatiotemporally adjacent pixel values in a subregion (or local window). The output of the neural filter is a single pixel value. The neural filter is trained with input images and corresponding “teaching” (desired or ideal) images. The training is performed by a linear-output backpropagation algorithm [40] which is a back-propagation algorithm modified for the linear-output ANN architecture. The input, output, and teacher (desired output) for neural filters are summarized in Table 2. Neural filters can acquire the functions of various linear and nonlinear filtering through training. Neural filters have been applied to reduction of the quantum noise in X-ray fluoroscopic and radiographic images [38, 39]. It was reported that the performance of the neural filter was superior to that of well-known nonlinear filters such as an adaptive weighted averaging filter [66]. A study [38] showed that adding features from the subregion to the input information improved the performance of the neural filter. Neural filters have been extended to accommodate the task of enhancement of edges, and a supervised edge enhancer (detector), called a neural edge enhancer, was developed [40]. The neural edge enhancer can acquire the function of a desired edge enhancer through training. It was reported that the performance of the neural edge enhancer in the detection of edges from noisy images was far superior to that of well-known edge detectors such as the Canny edge detector [67], the Marr-Hildreth edge detector [68], and the Huckel edge detector [69]. In its application to the contour extraction of the left ventricular cavity in digital angiography, it has been reported that the neural edge enhancer can accurately replicate the subjective edges traced by a cardiologist [41].

### 2.3. Massive-Training Artificial Neural Network (MTANN)

An MTANN was developed by extension of neural filters to accommodate various pattern-recognition tasks [52]. A two-dimensional (2D) MTANN was first developed for distinguishing a specific opacity (pattern) from other opacities (patterns) in 2D images [52]. The 2D MTANN was applied to reduction of FPs in computerized detection of lung nodules on 2D CT slices in a slice-by-slice way [17, 52, 63] and in CXR [57], the separation of ribs from soft tissue in CXR [54, 55, 70], and the distinction between benign and malignant lung nodules on 2D CT slices [58]. For processing of three-dimensional (3D) volume data, a 3D MTANN was developed by extending the structure of the 2D MTANN, and it was applied to 3D CT colonography data [53, 59–62].

**Figure 1:**
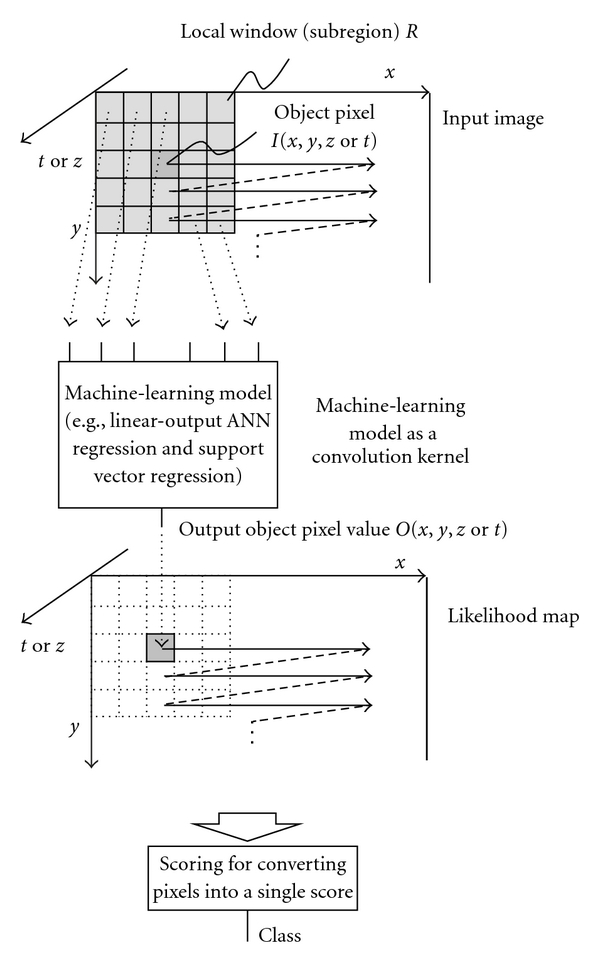
Generalized architecture of an MTANN (a class of PML) consisting of an ML model (e.g., linear-output ANN regression and support vector regression) with subregion input and single-pixel output. All pixel values in a subregion extracted from an input image are entered as input to the ML model. The ML model outputs a single pixel value for each subregion, the location of which corresponds to the center pixel in the subregion. Output pixel value is mapped back to the corresponding pixel in the output image.

The generalized architecture of an MTANN which unifies 2D and 3D MTANNs is shown in Figure 1. The input, output, and teacher for MTANNs are shown in Table 2. An MTANN consists of an ML model such as a linear-output ANN regression model and a support vector regression model, which is capable of operating on pixel/voxel data directly [40]. The linear-output ANN regression model employs a linear function instead of a sigmoid function as the activation function of the unit in the output layer because the characteristics of an ANN were improved significantly with a linear function when applied to the continuous mapping of values in image processing [40]. Note that the activation functions of the units in the hidden layer are a sigmoid function for nonlinear processing, and those of the unit in the input layer are an identity function, as usual. The pixel/voxel values of the input images/volumes may be normalized from 0 to 1. The input to the MTANN consists of pixel/voxel values in a subregion/subvolume, *R*, extracted from an input image/volume. The output of the MTANN is a continuous scalar value, which is associated with the center voxel in the subregion, and is represented by

(1) $$\begin{array}{cc} & O\left(x,y,z\;\text{or}\;t\right)\\ & \;=\text{ML}\left\{I\left(x-i,y-j,z-k\;\text{or}\;t-k\right)\;|\;\left(i,j,k\right)\in R\right\}, \end{array}$$

where *x*, *y*, and *z* or *t* are the coordinate indices, ML(*·*) is the output of the ML model, and *I*(*x*, *y*, *z*  or  *t*) is a pixel/voxel value of the input image/volume. A three-layer structure may be selected as the structure of the ANN, because it has been proved that any continuous mapping can be approximated by a three-layer ANN [71, 72]. The structure of input units and the number of hidden units in the ANN may be designed by use of sensitivity-based unit-pruning methods [73, 74]. Other ML models such as support vector regression [10, 11] can be used as a core part of the MTANN. ML regression models rather than ML classification models would be suited for the MTANN framework, because the output of the MTANN is continuous scalar values (as opposed to nominal categories or classes). The entire output image/volume is obtained by scanning with the input subvolume of the MTANN on the entire input image/volume. The input subregion/subvolume and the scanning with the MTANN can be analogous to the kernel of a convolution filter and the convolutional operation of the filter, respectively.

**Figure 2:**
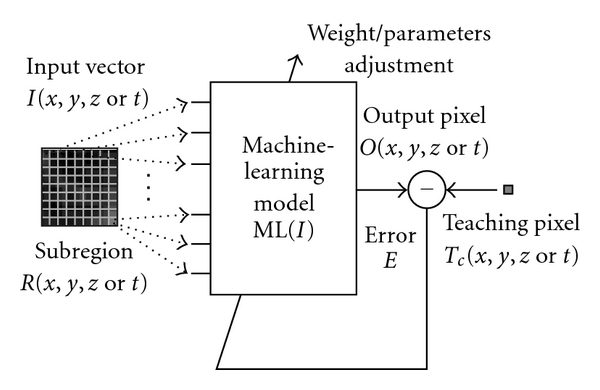
Training of an MTANN (a class of PML). An input vector is entered as input to the ML model. An error is calculated by subtracting of a teaching pixel from an output pixel. The parameters such as weights between layers in an ANN model are adjusted so that the error becomes small.

The training of an MTANN is shown in Figure 2. The MTANN is trained with input images/volumes and the corresponding “teaching” images/volumes for enhancement of a specific pattern and suppression of other patterns in images/volumes. The “teaching” images/volumes are ideal or desired images for the corresponding input images/volumes. For enhancement of lesions and suppression of nonlesions, the teaching volume contains a map for the “likelihood of being lesions,” represented by

(2) $$T\left(x,y,z\;\text{or}\;t\right)=\{\begin{array}{cc} \text{a}\;\text{certain}\;\text{distribution}, & \text{for}\;\text{a}\;\text{lesion},\\ 0, & \text{otherwise}. \end{array}$$

To enrich the training samples, a training region, *R*_*T*_, extracted from the input images is divided pixel by pixel into a large number of overlapping subregions. Single pixels are extracted from the corresponding teaching images as teaching values. The MTANN is massively trained by use of each of a large number of input subregions together with each of the corresponding teaching single pixels, hence the term “massive-training ANN.” The error to be minimized by training of the MTANN is represented by

(3) $$E=\frac{1}{P}\overset{}{\underset{c}{\sum }}\overset{}{\underset{\left(x,y,z\;\text{or}\;t\right)\in R_{T}}{\sum }}{\left\{T_{c}\left(x,y,z\;\text{or}\;t\right)-O_{c}\left(x,y,z\;\text{or}\;t\right)\right\}}^{2},$$

where *c* is a training case number, *O*_*c*_ is the output of the MTANN for the *c*th case, *T*_*c*_ is the teaching value for the MTANN for the *c*th case, and *P* is the number of total training voxels in the training region for the MTANN, *R*_*T*_. The expert 3D MTANN is trained by a linear-output backpropagation (BP) algorithm [40] which was derived for the linear-output ANN model by use of the generalized delta rule [8]. After training, the MTANN is expected to output the highest value when a lesion is located at the center of the subregion of the MTANN, a lower value as the distance from the subregion center increases, and zero when the input subregion contains a nonlesion.

A scoring method is used for combining output pixels from the trained MTANNs. A score for a given region of interest (ROI) from the MTANN is defined as

(4) $$S=\overset{}{\underset{\left(x,y,z\;\text{or}\;t\right)\in R_{E}}{\sum }}f_{W}\left(x,y,z\;\text{or}\;t\right)\times O\left(x,y,z\;\text{or}\;t\right),$$

where *f*_*W*_ is a weighting function for combining pixel-based output responses from the trained MTANN into a single score, which may often be the same distribution function used in the teaching images, and with its center corresponding to the center of the region for evaluation, *R*_*E*_; and *O* is the output image of the trained MTANN, where its center corresponds to the center of *R*_*E*_. This score represents the weighted sum of the estimates for the likelihood that the ROI (e.g., a lesion candidate) contains a lesion near the center; that is, a higher score would indicate a lesion and a lower score would indicate a non-lesion. Thresholding is then performed on the scores for distinction between lesions and non-lesions.

### 2.4. Convolution Neural Network (NN)

**Figure 3:**
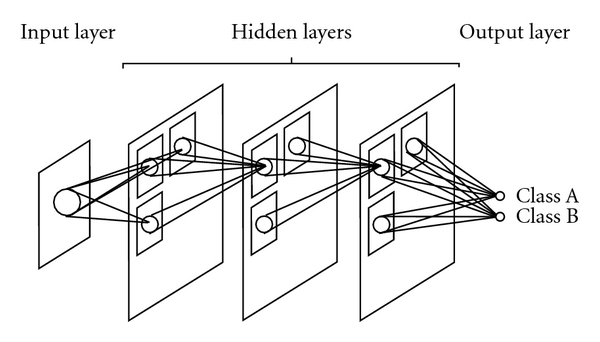
Architecture of a convolution NN (a class of PML). The convolution NN can be considered as a simplified version of the Neocognitron model, which was proposed to simulate the human visual system. The layers in the convolution NN are connected with local shift-invariant inter-connections (or convolution with a local kernel). The input and output of the convolution NN are images and nominal class labels (e.g., Class A and Class B), respectively.

A convolution NN has first been proposed for handwritten ZIP-code recognition [75]. The architecture of a convolution NN is illustrated in Figure 3. The input, output, and teacher for convolution NNs are summarized in Table 2. The convolution NN can be considered as a simplified version of the Neocognitron model [76–78] which was proposed to simulate the human visual system in 1980 [78]. The input and output of the convolution NN are images and nominal class labels, respectively. The convolution NN consists of one input layer, several hidden layers, and one output layer. The layers are connected with local shift-invariant interconnections (or convolution with a local kernel). Unlike the neocognitron, the convolution NN has no lateral interconnections or feedback loops, and the error BP algorithm [8] is used for training of the convolution NN. Units (neurons) in any hidden layer are organized in groups. Each unit in a subsequent layer is connected with the units of a small region in each group in the preceding layer. The groups between adjacent layers are interconnected by weights that are organized in kernels. For obtaining the shift-invariant responses, connection weights between any two groups in two layers are constrained to be shift-invariant; in other words, forward signal propagation is similar to a shift-invariant convolution operation. The signals from the units in a certain layer are convolved with the weight kernel, and the resulting value of the convolution is collected into the corresponding unit in the subsequent layer. This value is further processed by the unit through an activation function and produces an output signal. The activation function between two layers is a sigmoid function. For deriving the training algorithm for the convolution NN, the generalized delta rule [8] is applied to the architecture of the convolution NN. For distinguishing an ROI containing a lesion from an ROI containing a non-lesion, a class label (e.g., 1 for a lesion, 0 for a non-lesion) is assigned to an output unit.

Variants of the convolution NN have been proposed. The dual-kernel approach, which employs central kernels and peripheral kernels in each layer [43], was proposed for distinction between lung nodules and nonnodules in chest radiographs [42, 43] and distinction between microcalcifications and other anatomic structures in mammograms [43]. This dual-kernel-based convolution NN has several output units (instead of one or two output units in the standard convolution NN) for two-class classification. The fuzzy association was employed for transformation of output values from the output units to two classes (i.e., nodules or nonnodules; microcalcifications or other anatomic structures). A convolution NN which has subsampling layers has been developed for face recognition [47]. Some convolution NNs have one output unit [48, 79], some have two output units [80], and some have more than two output units [42, 43, 45, 47] for two-class classification.

Shift-invariant NNs [50, 51] are mostly the same as convolution NNs except for the output layer, which outputs images instead of classes. The shift-invariant NNs were used for localization (detection) of lesions in images, for example, detection of microcalcifications in mammograms [50, 51] and detection of the boundaries of the human corneal endothelium in photomicrographs [81].

### 2.5. Multilayer Perceptron for Character Recognition

**Figure 4:**
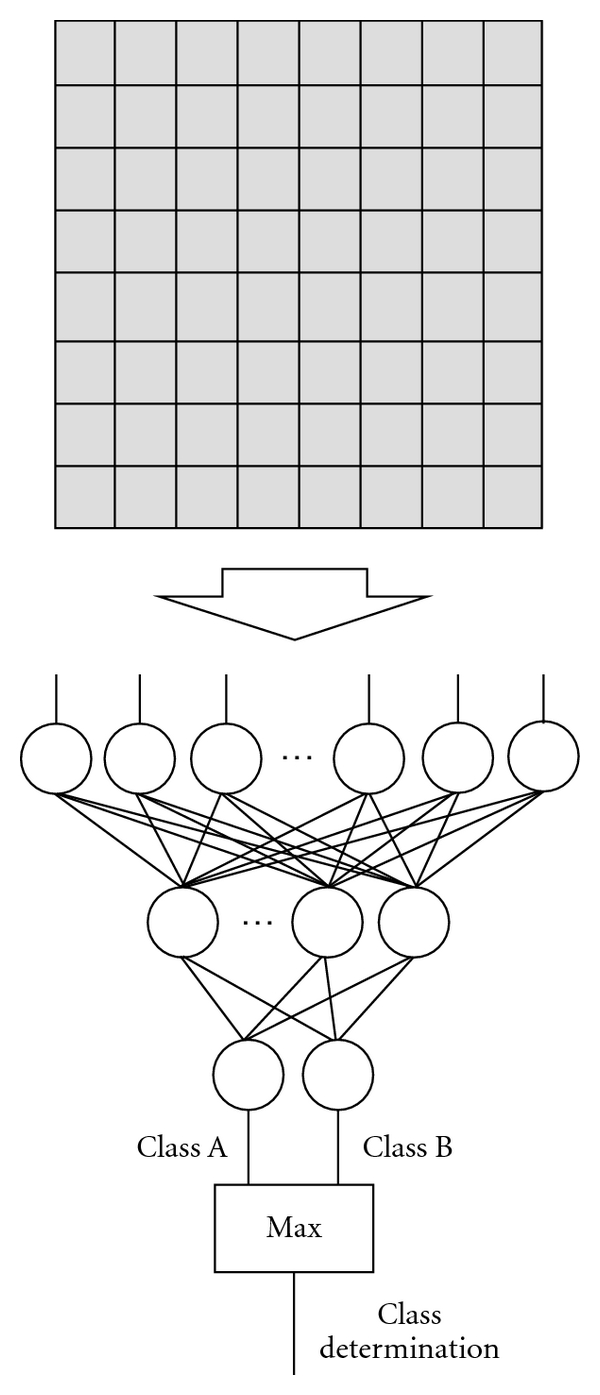
Architecture of a multilayer perceptron for character recognition. The binary pixel values in an image are entered as input to the multilayer perceptron. The class to which the given image belongs is determined as the class of the output unit with the maximum value.

A multilayer perceptron has been proposed for character recognition from an optical card reader [82, 83]. The architecture of the multilayer perceptron for character recognition is shown in Figure 4. The input, output, and teacher for the multilayer perceptron for character recognition are summarized in Table 2. The input and output of the multilayer perceptron are pixel values in a given binary image that contains a single character (e.g., a, b, or c) and a class to which the given image belongs, respectively. The number of input units equals the number of pixels in the given binary image (e.g., 16 × 16 pixels). The number of output units equals the number of classes (i.e., 26 for small-letter alphabetic characters). Each output unit is assigned to one of the classes. The class to which the given image belongs is determined as the class of the output unit with the maximum value. In the teaching data, a class label of 1 is assigned the corresponding output unit when a training sample belongs to that class; 0 is assigned to the other output units. Characters in given images are geometrically normalized before they are entered to the multilayer perceptron, because the architecture is not designed for being scale-invariant. Because character recognition with this type of the multilayer perceptron architecture is not shift-, rotation-, or scale-invariant, a large number of training samples is generally required. To enrich training samples, shifting, rotating, and scaling of training characters are often performed.

This type of multilayer perceptron has been applied to the classification of microcalcifications in mammography [23]. In this application, input images are not binary but gray-scale images. Pixel values in ROIs in mammograms or those in the Fourier-transformed ROIs were entered as input to the multilayer perceptron. In that study, the performance of the multilayer perceptrons based on ROIs in the spatial domain and the Fourier domain was found to be comparable.

### 2.6. Non-PML Feature-Based Classifiers

**Figure 5:**
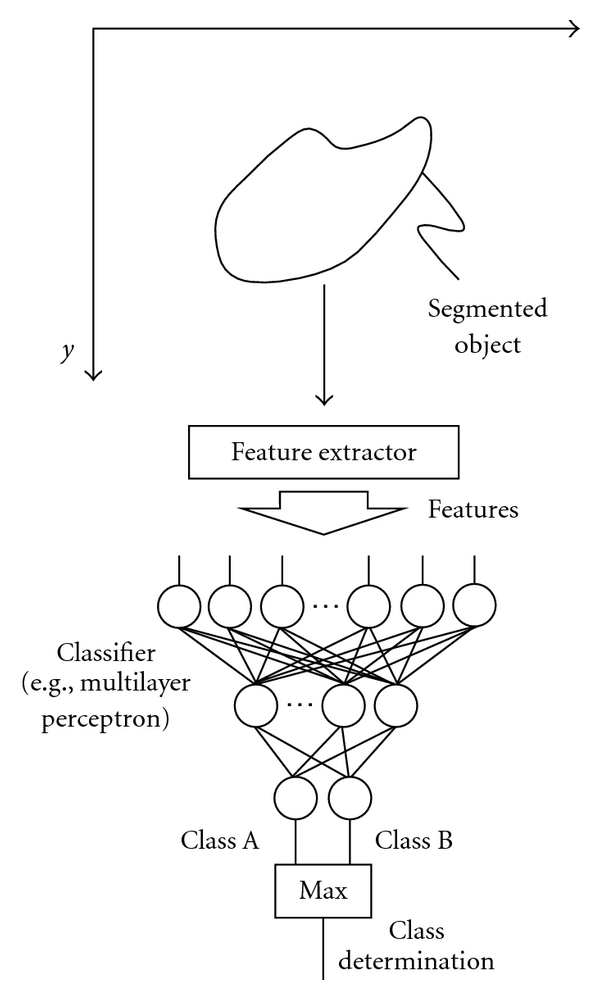
Standard classifier approach to classification of an object (i.e., feature-based ML). Features (e.g., contrast, effective diameter, and circularity) are extracted from a segmented object in an image. Those features are entered as input to a classifier such as a multilayer perceptron. Class determination is made by taking the class of the output unit with the maximum value.

One of most popular uses of ML algorithms would probably be classification. In this use, an ML algorithm is called a classifier. A standard classification approach based on a multilayer perceptron is illustrated in Figure 5. The input, output, and teacher for a classifier with features are summarized in Table 2. First, target objects are segmented by use of a segmentation method. Next, features are extracted from the segmented objects. Then, extracted features are entered as input to an ML model such as linear discriminant analysis [7], quadratic discriminant analysis [7], a multilayer perceptron [8, 9], and a support-vector machine [10, 11]. The ML model is trained with sets of input features and correct class labels. A class label of 1 is assigned to the corresponding output unit when a training sample belongs to that class, and 0 is assigned to the other output units. After training, the class of the unit with the maximum value is determined to be the corresponding class to which an unknown sample belongs. For details of feature-based classifiers, refer to one of many textbooks in pattern recognition such as [6–8, 10, 84].

## 3. Similarities and Differences

### 3.1. Within Different PML Algorithms

MTANNs [52] were developed by extension of neural filters to accommodate various pattern-recognition tasks. In other words, neural filters are a subclass or a special case of MTANNs. The applications and functions of neural filters are limited to noise reduction [38, 39] and edge enhancement [40, 41], whereas those of MTANNs were extended to include classification [52–54, 57–62], pattern enhancement and suppression [54], and object detection [56]. The input information to MTANNs, which is the pixel values in a subregion, is the same as that to neural filters. However, the output of (thus, teacher for) neural filters is the desired pixel values in a given image, whereas that of MTANNs is a map for the likelihood of being a specific pattern in a given image, as summarized in Table 2.

Both convolution NNs and the perceptron used for character recognition are in the class of PML. Input information to the convolution NNs and the perceptron is the pixel values in a given image, whereas the output of (thus, teacher for) both algorithms is a nominal class label for the given image. Thus, the input and output information are the same for both algorithms. However, the input images for the perceptron for character recognition are limited to be binary, although the perceptron itself is capable of processing gray-scale images. The major difference between convolution NNs and the perceptron used for character recognition is their internal architectures. Units in layers of the perceptron are fully connected, whereas the connections in the convolution NN are spatially (locally) limited. Because of this architecture, forward signal propagation in the convolution NN is realized by a convolution operation. This convolution operation offers a shift-invariant property which is desirable for image classification. The applications and functions of the perceptron are limited to character recognition such as zip code recognition and optical character recognition, whereas those of convolution NNs are general classification of images into known classes such as classification of lesion candidates into lesions or nonlesions [42–46], classification of faces [47], and classification of characters [48].

Shift-invariant NNs are mostly the same as convolution NNs except for the output layer, which outputs images instead of classes. The shift-invariant NNs can be used for localization (detection) of objects in images in addition to classification [50, 51].

Convolution NNs, shift-invariant NNs, and MTANNs perform convolution operations. In convolution NNs and shift-invariant NNs, convolution operations are performed within the network, as shown in Figure 3, whereas the convolutional operation is performed outside the network in the MTANN, as shown in Figure 1.

### 3.2. Between PML Algorithms and Ordinary Classifiers

The major difference between PMLs and ordinary classifiers (i.e., feature-based classifiers) is the input information. Ordinary classifiers use features extracted from a segmented object in a given image, whereas PMLs use pixel values in a given image as the input information. Although the input information to PMLs can be features (see addition of features to the input information to neural filters in [38], i.e.), these features are obtained pixel by pixel (rather than by object). In other words, features for PMLs are features at each pixel in a given image, whereas features for ordinary classifiers are features from a segmented object. In that sense, feature-based classifiers may be referred to as object-based classifiers. Because PMLs use pixel/voxel values in images directly instead of features calculated from segmented objects as the input information, feature calculation or segmentation is not required. Although the development of segmentation techniques has been studied for a long time, segmentation of objects is still challenging, especially for complicated objects, subtle objects, and objects in a complex background. Thus, segmentation errors may occur for complicated objects. Because, with PMLs, errors caused by inaccurate feature calculation and segmentation can be avoided, the performance of PMLs can be higher than that of ordinary classifiers for some cases, such as complicated objects.

The output information from ordinary classifiers, convolution NNs, and the perceptron used for character recognition is nominal class labels, whereas that from neural filters, MTANNs, and shift-invariant NNs is images. With the scoring method in MTANNs, output images of the MTANNs are converted to likelihood scores for distinguishing among classes, which allow MTANNs to do classification. In addition to classification, MTANNs can perform pattern enhancement and suppression as well as object detection, whereas the other PMLs cannot.

## 4. Applications of PML Algorithms in Medical Images

### 4.1. Edge-Preserving Noise Reduction by Use of Neural Filters

**Figure 6:**
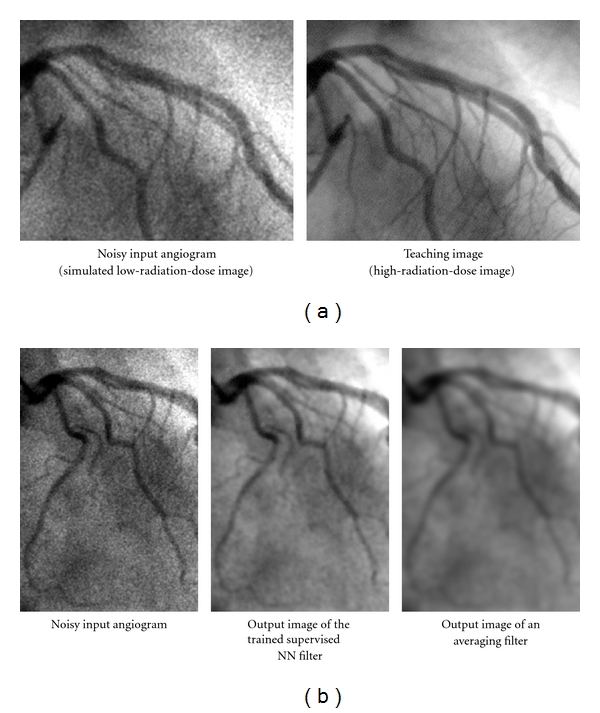
Reduction of quantum noise in angiograms by using a supervised NN filter called a “neural filter.” (a) Images used for training of the neural filter. (b) Result of an application of the trained neural filter to a nontraining image and a comparison result with an averaging filter.

Quantum noise is dominant in low-radiation-dose X-ray images used in diagnosis. For training a neural filter to reduce quantum noise in diagnostic X-ray images while preserving image details such as edges, noisy input images and corresponding “teaching” images are necessary. When a high radiation dose is used, X-ray images with little noise can be acquired and used as the “teaching” images. A noisy input image can be synthesized by addition of simulated quantum noise (which is modeled as signal-dependent noise) to a noiseless original high-radiation-dose image *f*_*o*_(*x*, *y*), represented by

(5) $$f_{N}\left(x,y\right)=f_{o}\left(x,y\right)+n\left[\sigma \left\{f_{o}\left(x,y\right)\right\}\right],$$

where *n*[*σ*{*f*_*o*_(*x*, *y*)}] is noise with standard deviation $\sigma \left\{f_{o}\left(x,y\right)\right\}=k_{N}\sqrt{f_{o}\left(x,y\right)}$ and *k*_*N*_ is a parameter determining the amount of noise. A synthesized noisy X-ray image obtained with this method and a noiseless original high-radiation-dose X-ray image are illustrated in Figure 6(a). They are angiograms of coronary arteries. They were used as the input image and as the teaching image for training of a neural filter. For sufficient reduction of noise, the input region of the neural filter consisted of 11 × 11 pixels. For efficient training of the entire image, 5,000 training pixels were sampled randomly from the input and teaching images. The training of the neural filter was performed for 100,000 iterations. The output image of the trained neural filter for a nontraining case is shown in Figure 6(b). The noise in the input image is reduced while image details such as the edges of arteries are maintained. When an averaging filter was used for noise reduction, the edges of arteries were blurry, as shown in Figure 6(b).

### 4.2. Edge Enhancement from Noisy Images by Use of Neural Edge Enhancer

**Figure 7:**
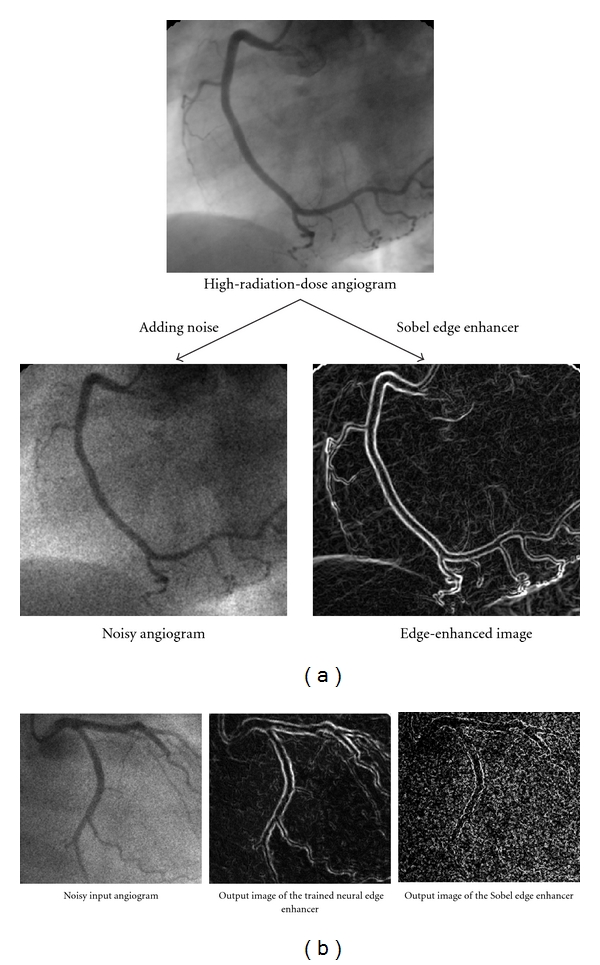
Enhancement of edges from noisy images by use of a supervised edge enhancer called a “neural edge enhancer.” (a) A way to create noisy input images and corresponding “teaching” edge images from noiseless images for training a neural edge enhancer. (b) Result of an application of the trained neural edge enhancer to a nontraining image and a comparison result with a Sobel edge enhancer.

Although conventional edge enhancers can very well enhance edges in images with little noise, they do not work well on noisy images. To address this issue, a neural edge enhancer has been developed for enhancing edges from very noisy images [40]. The neural edge enhancer is based on a neural filter and can be trained with input images and corresponding “teaching” edge images.  Figure 7(a) shows a way of creating noisy input images and corresponding “teaching” edge images from a noiseless image for training of a neural edge enhancer. Simulated quantum noise was added to original noiseless images to create noisy input images. A Sobel edge enhancer [85] was applied to the original noiseless images to create “teaching” edge images. The key here is that the Sobel edge enhancer works very well for noiseless images. The neural edge enhancer was trained with the noisy input images together with the corresponding teaching edge images. For comparison, the trained neural edge enhancer and the Sobel edge enhancer were applied to nontraining noisy images. The resulting nontraining edge-enhanced images are shown in Figure 7(b). Edges are enhanced clearly in the output image of the neural edge enhancer while noise is suppressed, whereas the Sobel edge enhancer enhances not only edges but also noise.

### 4.3. Bone Separation from Soft Tissue in Chest Radiographs (CXRs) by Use of MTANNs

CXR is the most frequently used diagnostic imaging examination for chest diseases such as lung cancer, tuberculosis, and pneumonia. More than 9 million people worldwide die annually from chest diseases [86]. Lung cancer causes 945,000 deaths and is the leading cause of cancer deaths in the world [86] and in countries such as the United States, the United Kingdom, and Japan [87]. Lung nodules (i.e., potential lung cancers) in CXR, however, can be overlooked by radiologists in from 12 to 90% of cases that have nodules visible in retrospect [88, 89]. Studies showed that 82 to 95% of the missed lung cancers were partly obscured by overlying bones such as ribs and/or a clavicle [88, 89]. To address this issue, dual-energy imaging has been investigated [90, 91]. Dual-energy imaging uses the energy dependence of the X-ray attenuation by different materials; it can produce two tissue-selective images, that is, a “bone” image and a “soft-tissue” image [92–94]. Major drawbacks of dual-energy imaging, however, are that (a) the radiation dose can be double, (b) specialized equipment for obtaining dual-energy X-ray exposures is required, and (c) the subtraction of two-energy images causes an increased noise level in the images.

**Figure 8:**
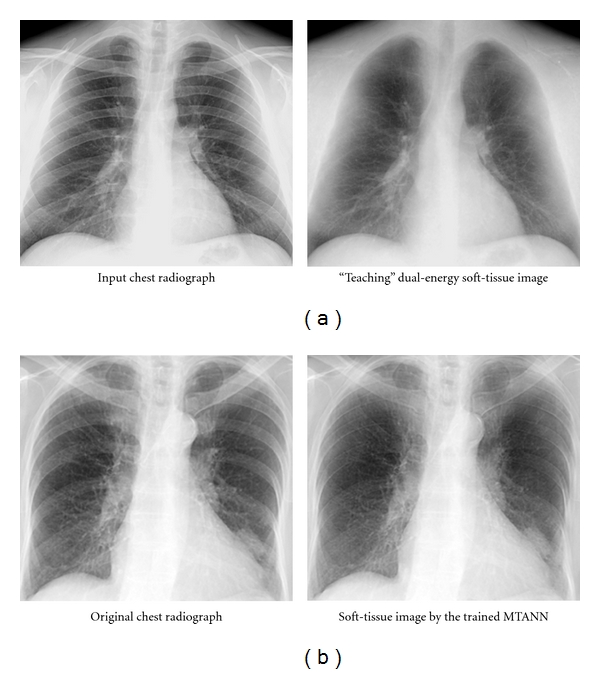
Separation of bones from soft tissue in CXRs by use of an MTANN. (a) Images used for training the MTANN. (b) Result of an application of the trained MTANN to a nontraining CXR.

For resolving the above drawbacks with dual-energy images, MTANNs have been developed as an image-processing technique for separation of ribs from soft tissue [54, 70]. The basic idea is to train the MTANN with soft-tissue and bone images acquired with a dual-energy radiography system [92, 95, 96]. For separation of ribs from soft tissue, the MTANN was trained with input CXRs and the corresponding “teaching” dual-energy bone images, as illustrated in Figure 8(a).  Figure 8(b) shows a nontraining original CXR and a soft-tissue image obtained by use of the trained MTANN. The contrast of ribs is suppressed substantially in the MTANN soft-tissue image, whereas the contrast of soft tissue such as lung vessels is maintained. There is another PML approach called filter learning to do the same task [64].

### 4.4. Enhancement and Detection of Lesions by Use of MTANNs

Computer-aided diagnosis (CAD) has been an active area of study in medical image analysis [1, 2, 97, 98]. Some CAD schemes employ a filter for enhancement of lesions as a preprocessing step for improving sensitivity and specificity, but some do not employ such a filter. The filter enhances objects similar to a model employed in the filter; for example, a blob-enhancement filter based on the Hessian matrix enhances sphere-like objects [99]. Actual lesions, however, often differ from a simple model; for example, a lung nodule is generally modeled as a solid sphere, but there are nodules of various shapes and inhomogeneous nodules such as nodules with spiculation and ground-glass nodules. Thus, conventional filters often fail to enhance such actual lesions.

**Figure 9:**
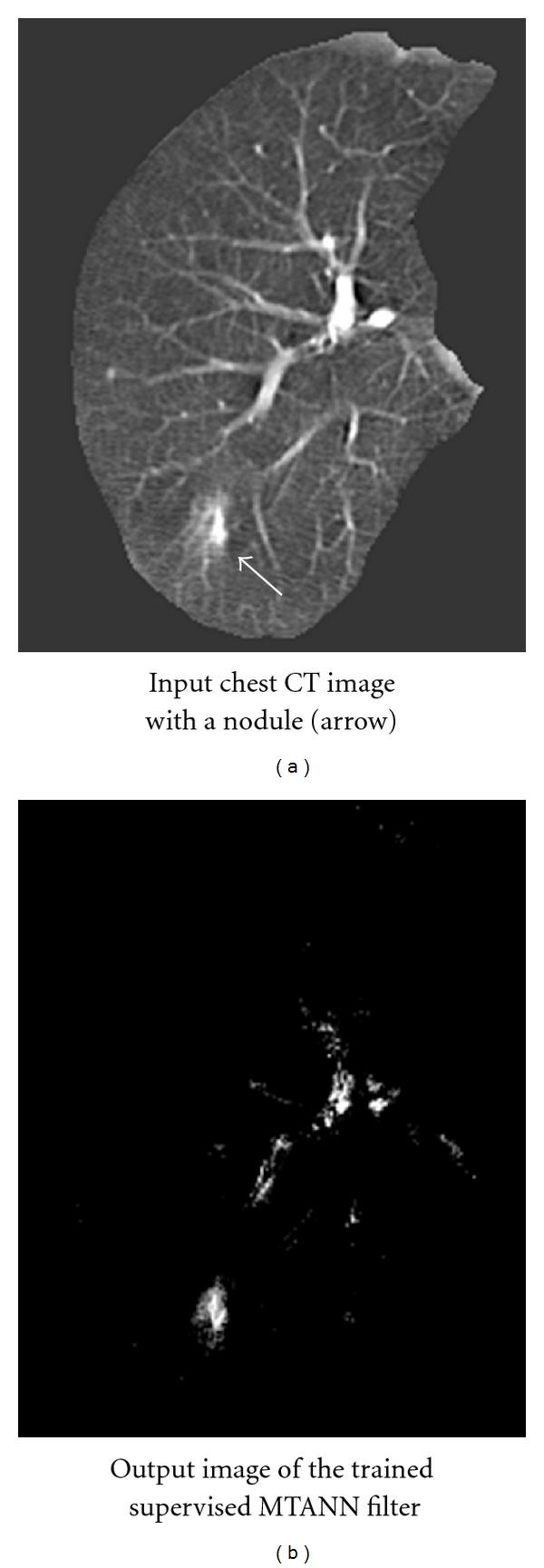
Enhancement of a lesion by use of the trained lesion-enhancement MTANN filter for a nontraining case. (a) Original chest CT image of the segmented lung with a nodule (indicated by an arrow). (b) Output image of the trained lesion-enhancement MTANN filter.

To address this issue, a “lesion-enhancement” filter based on MTANNs has been developed for enhancement of actual lesions in a CAD scheme for detection of lung nodules in CT [56]. For enhancement of lesions and suppression of non-lesions in CT images, the teaching image contains a map for the “likelihood of being lesions.” For enhancement of a nodule in an input CT image, a 2D Gaussian distribution was placed at the location of the nodule in the teaching image, as a model of the likelihood of being a lesion. For testing of the performance, the trained MTANN was applied to nontraining lung CT images. As shown in Figure 9, the nodule is enhanced in the output image of the trained MTANN filter, while normal structures such as lung vessels are suppressed. Note that small remaining regions due to vessels can easily be separated from nodules by use of their area information which can be obtained by use of connected-component labeling [100–102].

### 4.5. Classification between Lesions and Nonlesions by Use of Different PML Algorithms

#### 4.5.1. MTANNs

**Figure 10:**
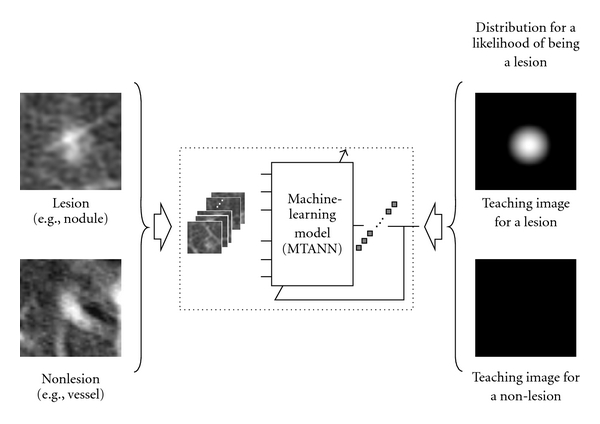
Training of an MTANN for distinction between lesions and non-lesions in a CAD scheme for detection of lesions in medical images. The teaching image for a lesion contains a Gaussian distribution; that for a non-lesion contains zero (completely dark). After the training, the MTANN expects to enhance lesions and suppress non-lesions.

A major challenge in CAD development is to reduce the number of FPs [27, 103–107], because there are various normal structures similar to lesions in medical images. To address this issue, an FP-reduction technique based on an MTANN has been developed for a CAD scheme for lung nodule detection in CT [52]. For enhancement of nodules (i.e., true positives) and suppression of nonnodules (i.e., FPs) on CT images, the teaching image contains a distribution of values that represent the “likelihood of being a nodule.” For example, the teaching volume contains a 3D Gaussian distribution with standard deviation *σ*_*T*_ for a lesion and zero (i.e., completely dark) for non-lesions, as illustrated in Figure 10. This distribution represents the “likelihood of being a lesion”:

(6) $$\begin{array}{cc} & T\left(x,y,z\;\text{or}\;t\right)\\ & \;=\{\begin{array}{cc} \frac{1}{\sqrt{2\pi }\sigma_{T}}\;\exp\left\{-\frac{\left(x^{2}+y^{2}+z^{2}\;\text{or}\;t^{2}\right)}{2\sigma_{T}^{2}}\right\}, & \text{for}\;\text{a}\;\text{lesion},\\ 0, & \text{otherwise}. \end{array} \end{array}$$

A 3D Gaussian distribution is used to approximate an average shape of lesions. The MTANN involves training with a large number of subvolume-voxel pairs, which is called a massive-subvolumes training scheme.

**Figure 11:**
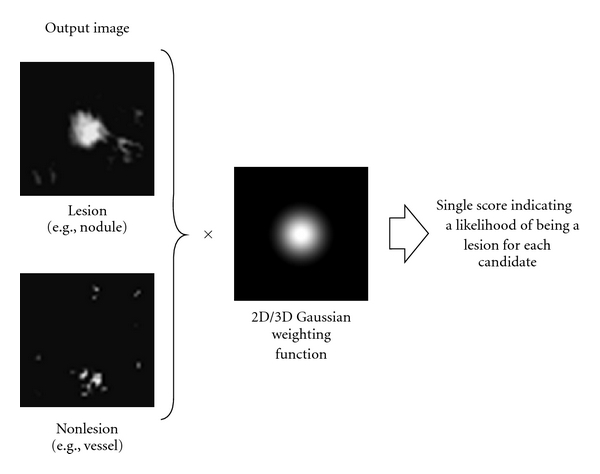
Scoring method for combining pixel-based output responses from the trained MTANN into a single score for each ROI.

A scoring method is used for combining of output voxels from the trained MTANNs, as illustrated in Figure 11. A score for a given ROI from the MTANN is defined as

(7) $$S=\overset{}{\underset{\left(x,y,z\;\text{or}\;t\right)\in R_{E}}{\sum }}f_{W}\left(x,y,z\;\text{or}\;t\right)\times O\left(x,y,z\;\text{or}\;t\right),$$

where

(8) $$\begin{array}{cc} & f_{W}\left(x,y,z\;\text{or}\;t\right)\\ & \;=f_{G}\left(x,y,z\;\text{or}\;t;\sigma \right)=\frac{1}{\sqrt{2\pi }\sigma }e^{-(x^{2}+y^{2}+z^{2}\;\text{or}\;t^{2})/2\sigma^{2}} \end{array}$$

is a 3D Gaussian weighting function with standard deviation *σ* and with its center corresponding to the center of the volume for evaluation, *R*_*E*_, and *O* is the output image of the trained MTANN, where its center corresponds to the center of *R*_*E*_. The use of the 3D Gaussian weighting function allows us to combine the responses (outputs) of a trained MTANN as a 3D distribution. A 3D Gaussian function is used for scoring, because the output of a trained MTANN is expected to be similar to the 3D Gaussian distribution used in the teaching images. This score represents the weighted sum of the estimates for the likelihood that the ROI (lesion candidate) contains a lesion near the center; that is, a higher score would indicate a lesion and a lower score would indicate a nonlesion. Thresholding is then performed on the scores for distinction between lesions and non-lesions.

**Figure 12:**
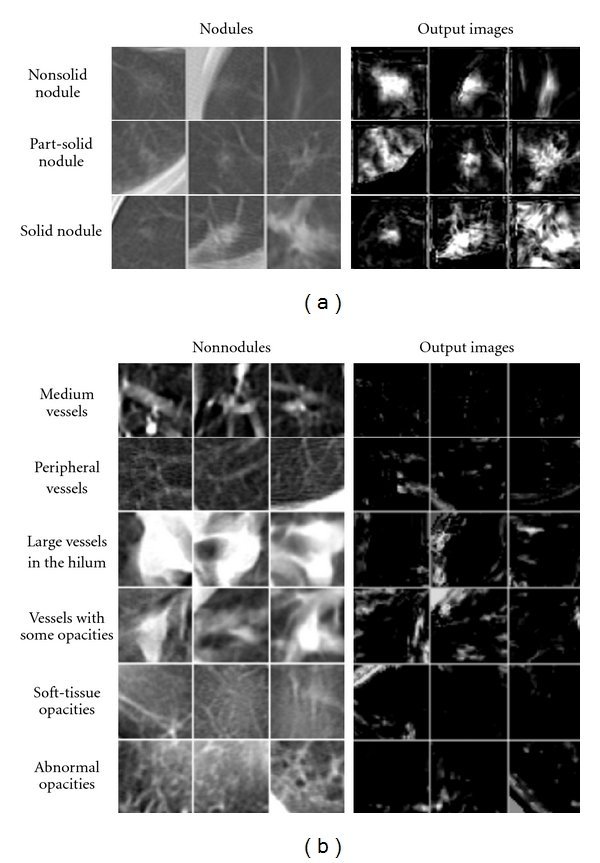
Illustrations of various types of nontraining nodules and nonnodules and corresponding output images of the trained MTANN. Nodules are represented by bright pixels, whereas nonnodules are almost dark around the centers of ROIs.

**Figure 13:**
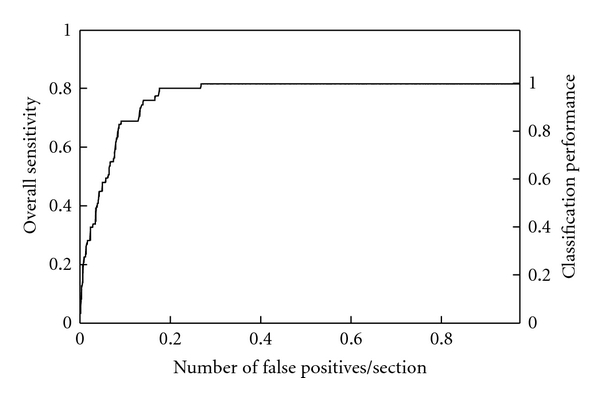
FROC curve indicating the performance of the MTANN in distinction between 57 true positives (nodules) and 1.726 FPs (nonnodules).

An MTANN was trained with typical nodules and typical types of FPs (nonnodules) and corresponding teaching images. The trained MTANN was applied to 57 true positives (nodules) and 1,726 FPs (nonnodules) produced by a CAD scheme [52].  Figure 12 shows various types of nodules and nonnodules and the corresponding output images of the trained MTANN. Nodules such as a solid nodule, a part-solid (mixed-ground-glass) nodule, and a non-solid (ground-glass) nodule are enhanced, whereas nonnodules such as different-sized lung vessels and soft-tissue opacity are suppressed around the centers of ROIs. For combining output pixels into a single score for each nodule candidate, a scoring method was applied to the output images for distinction between a nodules and a nonnodule. Thresholding of scores was done for classification of nodule candidates into nodules or nonnodules. Free-response receiver operating characteristic (FROC) analysis [108] was carried out for evaluation of the performance of the trained MTANN. The FROC curve for the MTANN indicates 80.3% overall sensitivity (100% classification performance) and a reduction in the FP rate from 0.98 to 0.18 per section, as shown in Figure 13.

#### 4.5.2. Convolution NNs and Shift-Invariant NNs

Convolution NNs have been used for FP reduction in CAD schemes for lung nodule detection in CXRs [42–44]. A convolution NN was trained with 28 chest radiographs for distinguishing lung nodules from nonnodules (i.e., FPs produced by an initial CAD scheme). The trained convolution NN reduced 79% of FP detections (which is equivalent to 2-3 FPs per patient), while 80% of true-positive detections were preserved. Convolution NNs have been applied to FP reduction in CAD schemes for detection of microcalcifications [45] and masses [46] in mammography. A convolution NN was trained with 34 mammograms for distinguishing microcalcifications from FPs. The trained convolution NN reduced 90% of FP detections, which resulted in 0.5 FP detections per image, while a true-positive detection rate of 87% was preserved [45].

Shift-invariant NNs have been used for FP reduction in CAD for detection of microcalcifications [50, 51]. A shift-invariant NN was trained to detect microcalcifications in ROIs. Microcalcifications were detected by thresholding of the output images of the trained shift-invariant NN. When the number of detected microcalcifications was greater than a predetermined number, the ROI was considered as a microcalcification ROI. With the trained shift-invariant NN, 55% of FPs was removed without any loss of true positives.

## 5. Advantages and Limitations of PML Algorithms

As described earlier, the major difference between PMLs and ordinary classifiers is the direct use of pixel values with PML. In other words, unlike ordinary classifiers, feature calculation from segmented objects is not necessary. Because the PML can avoid errors caused by inaccurate feature calculation and segmentation, the performance of the PML can potentially be higher than that of ordinary feature-based classifiers for some cases. PMLs learn pixel data directly, and thus all information on pixels should not be lost before the pixel data are entered into the PML, whereas ordinary feature-based classifiers learn the features extracted from segmented lesions and thus important information can be lost with this indirect extraction; also, inaccurate segmentation often occurs for complicated patterns. In addition, because feature calculation is not required for PML, development and implementation of segmentation and feature calculation, and selection of features are unnecessary.

Ordinary classifiers such as linear discriminant analysis, ANNs, and support vector machines cannot be used for image processing, detection (localization) of objects, or enhancement of objects or patterns, whereas MTANNs can do those tasks. For example, MTANNs can separate bones from soft tissue in CXRs [54], and MTANN can enhance and detect lung nodules on CT images [56].

The characteristics of PMLs which use pixel data directly should differ from those of ordinary feature-based classifiers. Therefore, combining an ordinary feature-based classifier with a PML would yield a higher performance than that of a classifier alone or a PML alone. Indeed, in previous studies, both classifier and PML were used successfully for classification of lesion candidates into lesions and non-lesions [17, 45, 46, 49–53, 58–63].

A limitation of PMLs is the relatively long time for training because of the high dimensionality of input data. Because PMLs use pixel data in images directly, the number of input dimensions is generally large. For example, a 3D MTANN for 3D CT data requires 171 dimensions for its input [53, 60]. The ordinary feature-based classifiers are more efficient than PMLs. In an application of PMLs and feature-based classifiers to CAD schemes, a feature-based classifier should be applied first, because the number of lesion candidates that need to be classified is larger at an earlier stage. After the number of lesion candidates is reduced by use of the feature-based classifier, a PML should be applied for further reduction of FPs. Indeed, previous studies employed this strategy [17, 52, 53, 58–61].

To address the issue of training time for PML, dimensionality reduction methods for PML have been proposed [61]. With the use of the Laplacian-eigenfunction-based dimensionality reduction of the input vectors to a 3D MTANN, the training time was reduced by a factor of 8.5.

## 6. Conclusion

In this paper, PMLs were surveyed and compared with each other as well as with other non-PML algorithms (i.e., ordinary feature-based classifiers) to make the similarities, differences, advantages, and limitations clear. The major difference between PMLs and non-PML algorithms (e.g., classifiers) is a need for segmentation and feature calculation with non-PML algorithms. The major advantage of PMLs over non-PML algorithms is that no information is lost due to inaccurate segmentation and feature calculation, which would result in a higher performance for some cases such as complicated patterns. With the combination of PMLs with non-PML algorithms, the performance of a system can be improved substantially. In addition to a classification task, MTANNs can be used for enhancement (and suppression) and detection (i.e., localization) of objects (or patterns) in images.

## Figures and Tables

**Table 1:** Classes of PMLs, their functions, and their applications.

PMLs	Functions	Applications
Neural filters (including neural edge enhancers)	Image processing	Edge-preserving noise reduction [38, 39]. Edge enhancement from noisy images [40]. Enhancement of subjective edges traced by a physician [41].
Convolution neural networks (including shift-invariant neural networks)	Classification	FP reduction in CAD for lung nodule detection in CXR [42–44]. FP reduction in CAD for detection of microcalcifications [45] and masses [46] in mammography. Face recognition [47]. Character recognition [48].
Massive-training artificial neural networks (MTANNs, including a mixture of expert MTANNs, a LAP-MTANN, an MTSVR)	Classification (image processing + scoring), pattern enhancement and suppression, object detection (pattern enhancement followed by thresholding or segmentation)	FP reduction in CAD for detection of lung nodules in CXR [57] and CT [17, 52, 63]. Distinction between benign and malignant lung nodules in CT [58]. FP reduction in CAD for polyp detection in CT colonography [53, 59–62]. Bone separation from soft tissue in CXR [54, 55]. Enhancement of lung nodules in CT [56].
Others	Image processing or classification	Segmenting posterior ribs in CXR [64]. Separation of ribs from soft tissue in CXR [65].

**Table 2:** Classification of ML algorithms by their input, output, and teacher (desired output).

ML algorithms	Input	Output	Teacher
Neural filters	Pixel values in a subregion (local window) in a given image	Single pixel value (image is formed by collecting pixels)	Desired pixel value
MTANNs	Pixel values in a subregion (local window) in a given image	Single pixel value (image is formed by collecting pixels; likelihood score for the given image is obtained by use of the scoring method)	Likelihood of being a specific pattern at each pixel
Convolution NNs	Pixel values in a given image	Class to which the given image belongs	Nominal class label for the given image
Shift-invariant NNs	Pixel values in a given image	Class to which each pixel belongs	Nominal class label for each pixel
Multilayer perceptron for character recognition	Pixel values in a given binary image (character)	Class to which the given image belongs	Nominal class label for the given image
Classifiers (e.g., linear discriminant analysis, NNs, support vector machines)	Features extracted from a segmented object in a given image	Class to which the segmented object belongs	Nominal class label for the segmented object
